# Lithography-free subwavelength metacoatings for high thermal radiation background camouflage empowered by deep neural network

**DOI:** 10.1515/nanoph-2025-0409

**Published:** 2025-12-08

**Authors:** Qianli Qiu, Kang Li, Dongjie Zhou, Yuyang Zhang, Jinguo Zhang, Zongkun Zhang, Yan Sun, Lei Zhou, Ning Dai, Junhao Chu, Jiaming Hao

**Affiliations:** State Key Laboratory of Infrared Physics, Shanghai Institute of Technical Physics, Chinese Academy of Science, Shanghai, 200083, China; University of Chinese Academy of Sciences, No. 19A Yu Quan Road, Beijing, 100049, China; Institute of Optoelectronics & College of Future Information Technology, Shanghai Frontiers Science Research Base of Intelligent Optoelectronics and Perception, and State Key Laboratory of Photovoltaic Science and Technology, 12478 Fudan University, Shanghai, 200433, China; Shanghai Key Laboratory of Metasurfaces for Light Manipulation, State Key Laboratory of Surface Physics, Key Laboratory of Micro and Nano Photonic Structures (Ministry of Education) and Department of Physics, Fudan University, Shanghai, 200433, China; Hangzhou Institute for Advanced Study, University of Chinese Academy of Sciences, Hangzhou, 310024, China

**Keywords:** LWIR camouflage, deep neural network, metacoating, deep-subwavelength

## Abstract

The long wavelength infrared (LWIR) range (8–14 µm) is crucial for thermal radiation detection, necessitating effective camouflage against advanced infrared technologies. Conventional camouflage approaches often rely on complicated photonic structures, facing significant implementation challenges. This study introduces a novel polarization-insensitive and angle-robust metacoating emitter for LWIR camouflage, inversely designed through a deep neural network (DNN) framework. The DNN framework facilitates the automatic optimization of the metacoating’s structural and material parameters. The resulting emitter achieves an average emissivity of 0.96 covering the LWIR range and a low emissivity of 0.25 in the other mid-infrared (MIR) region. Enhanced electromagnetic wave localization and energy dissipation, driven by high-lossy materials like bismuth and titanium, contribute to these properties. Infrared imaging confirms the emitter’s superior camouflage performance, maintain effectiveness at incident angle up to 70° while exhibiting strong polarization independence. This inverse-designed metacoating demonstrates significant potential to advance infrared camouflage technology, providing robust countermeasures against modern, wide-angle, and polarization-sensitive detection systems.

## 1 Introduction

Long wavelength infrared (LWIR) is a subset of the infrared band of the electromagnetic wave spectrum, covering the wavelengths ranging from 8 µm to 14 µm [1]. Electromagnetic radiation emitted by the objects on earth at the room temperature is mainly located in this range, which can pass through the earth’s atmosphere without distortion or absorption [2], [3], [4], [5], [6], and can be detected by infrared equipment [7]. Therefore, with the rapid development of infrared detection technology [8], [9], [10], it is of great significance to investigate novel counter-detection technology to control the absorption and emission of objects in the LWIR [11], [12]. According to the Stefan–Boltzmann law, the emission energy of an object is related to its absolute temperature and emissivity. Hence, when the temperature of the concealed target reaches equilibrium with that of the background, an effective way to regulate the thermal radiation of the target for thermal camouflage is to manipulate the emissivity of the target [13], [14].

To achieve effective thermal camouflage under high-emission environments, selective emitters must exhibit specific radiative properties. Particularly, they ought to demonstrate broadband high emissivity within the 8–14 µm LWIR spectral range and low emissivity in other regions [15]. In recent decades, artificial optical structures such as photonic crystals (PCs) [16], [17], [18], metasurfaces (MSs) [19], [20], [21], [22], and metamaterials (MMs) [23], [24], [25], [26], [27], [28] have showcased the ability to manipulate electromagnetic (EM) waves and successfully realize these desired emission characteristics. However, the fabrication of these subwavelength structures, particularly PCs and MSs, often relies on high-precision techniques such as electron-beam lithography and focused ion beam milling. Although these methods enable intricate nanoscale patterning, they are typically time-consuming, costly, and impose strict requirements on substrate smoothness. These drawbacks lead to inefficient production, which ultimately hinders the practical implementation and scalability of advanced LWIR camouflage devices for real-world applications [29].

The approach of multilayered metacoating (MMC) by depositing thin-film materials with diverse optical properties onto target surfaces provides a cost-effective means of tailoring thermal emission characteristics [30], [31], [32], [33], [34]. This methodology offers flexibility and adaptability in tuning the surface emission features to meet specific requirements for thermal camouflage applications [35], [36], [37], [38], [39], [40]. However, while numerous material combinations for metacoatings have been proposed to modulate infrared emission, both material selection and structural design remain heavily dependent on physics-inspired methods and past design experience or guidelines [41], [42]. This inherently inefficient approach poses challenges in achieving the optimal design to meet the ideal spectra. This trial-and-error approach can be inefficient and may hinder the achievement of optimal designs that align with desired spectral profiles [43], [44]. To overcome these limitations, it is meaningful to develop intelligent design method for automatic meeting the required emissivity without such constraints [45], [46], [47], [48]. While data-driven deep learning has demonstrated exceptional efficacy in multi-physics optimization, its application to emissivity engineering faces two fundamental bottlenecks: (i) an obligatory dependence on expansive, pre-computed training datasets, and (ii) the a-prior specification of material constituents and layer thicknesses, which constrains the explorable design space and precludes genuinely knowledge-free optimization.

In this study, we present the design and experimental validation of an angle-robust, deep-subwavelength broadband metacoating for LWIR camouflage in high-emission environments. Our approach leverages a deep neural network (DNN) framework that automates the optimization of structural parameters and material selection to achieve a target spectral response. A key innovation is the direct integration of the DNN with TMM calculations, enabling an efficient, data-free optimization workflow that operates without pre-trained models or large datasets. The optimized metacoating exhibits an exceptional average emissivity of 0.96 across the LWIR atmospheric transmission window (ATW), while maintaining a low emissivity of 0.25 in the adjacent mid-infrared (MIR) spectral region, as corroborated by both experimental and theoretical analyses. The outstanding thermal emission characteristics of the metacoating are primarily attributed to its excellent electromagnetic localization ability, facilitated by the multilayer structure, and the subsequent localization-induced energy dissipation via nearby high-lossy materials, particularly bismuth and titanium. Infrared imaging experiments further demonstrate the superior camouflage performance of the proposed emitter against highly radiative backgrounds. Notably, the metacoating structure exhibits angle-independent emission properties, with favorable emissive behavior observed at high incidence angles up to 70°. The angular robustness and polarization insensitivity properties of the device offer a significant advantage in LWIR camouflage scenarios, where modern detection methods often employ wide-angle and polarization-sensitive measurements. In conclusion, our results highlight the substantial promise of the developed metacoating emitter for advancing the state-of-the-art in IR camouflage technology.

## 2 Results and discussion

**Figure 1:**
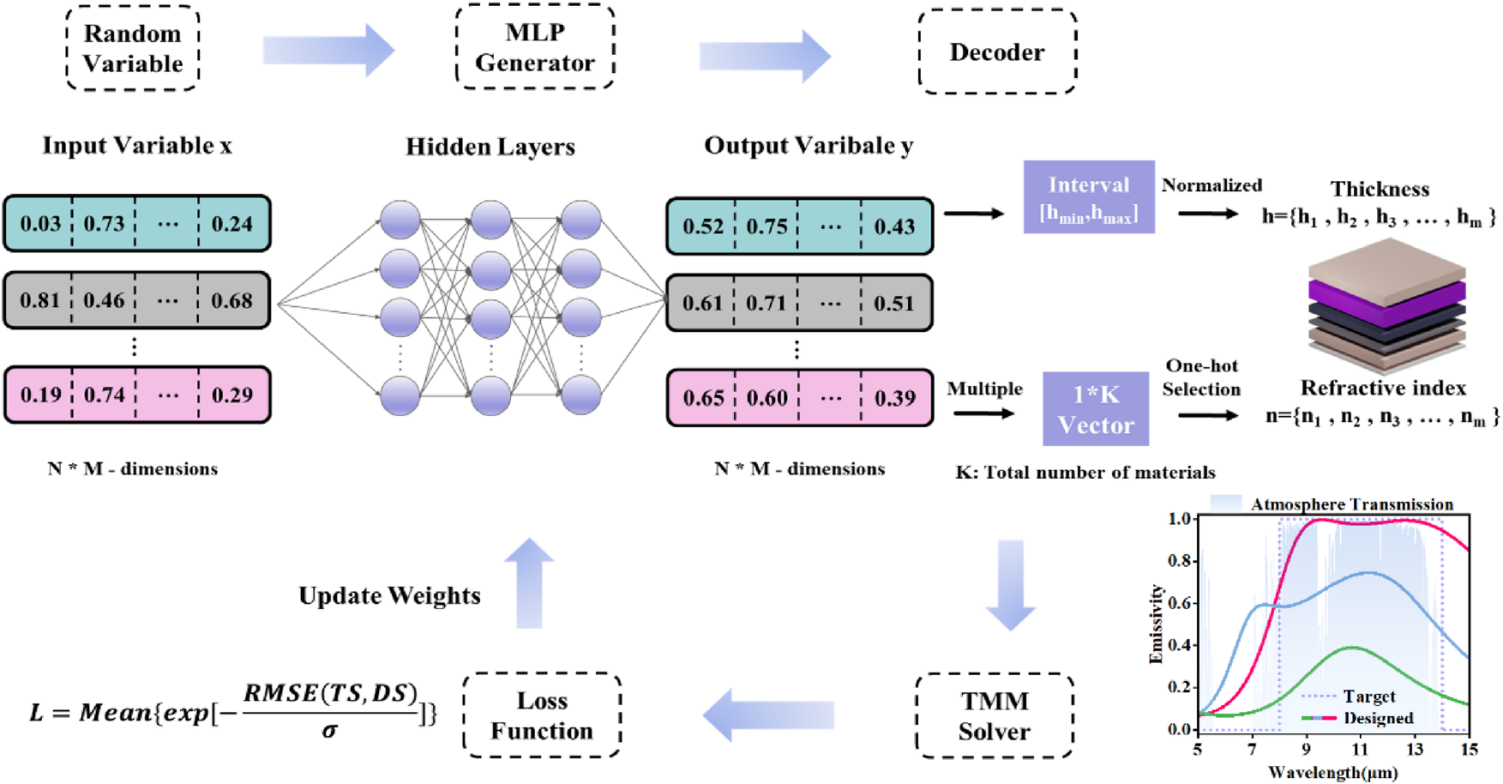
Schematic of the inversed design process for the subwavelength metacoatings. A multilayer perceptron (MLP) generator maps a random variable distribution to a distribution of device with varying thickness and materials by a decoder using predefined rules, evaluated using a transfer matrix method (TMM) solver, and the resulting loss function is used to update the network weights through backpropagation, enabling the optimization to the setting targets.

The optimization framework for DNN targeting high-emissivity background camouflage in the LWIR range is illustrated in Figure 1. This approach comprises three core components: the multilayer perceptron (MLP) generator [49], the decoder, and the transfer matrix method (TMM) solver. Initially, random variables are generated and input into an untrained MLP. The outputs are then processed by the decoder, which translates them into the distribution characteristics of our proposed metacoating device, varying in structure, thickness, and material. A key design constraint is the limitation to 20 layers, each 20–300 nm thick, which guarantees the deep-subwavelength nature of the device. This property is critical for minimizing phase variance and thus improving angle robustness in the LWIR spectrum. The material for each random MMC device is selected using one-hot encoding from a database of commonly used materials, including metals (Au, Ag, Al, Ti, Cr), semi-metal (Bi) and dielectrics (YbF3, MgF2, Si, Ge, ZnS, SiO2, HfO2). The TMM solver calculates the emissivity spectra (*E*) of the MMC devices using the formula *E* = *A* = *1* - *R* - *T*, where *A* is absorbance, *R* is reflectance, and *T* is transmittance. The design spectra (DS), calculated by the TMM solver across the 3–14 μm range, are evaluated against a target spectrum (TS). The TS is defined by a prescribed emissivity profile: zero emissivity (*ε* = 0) within the 3–8 μm wavelength range and unit emissivity (*ε* = 1) within the 8–14 μm atmospheric window. Both spectra are discretized over the 3–14 μm range with a uniform spectral interval of 100 nm. A loss function is constructed as:

$$L=\text{Mean}\left(\exp\left(-\frac{\text{RMSE}\left(\text{TS,DS}\right)}{\sigma }\right)\right),$$

σ serving as a convergence-enhancing hyperparameter. Gradients of the loss function are calculated using the chain rule, and adjustment of the MLP weights is guided by Adam method [47] (the network parameter settings and testing results are detailed in Section S1 of the supplementary material). This iterative training process continues until a predefined stopping condition is met. Upon completion, the DNN can output optimized MMC devices closely matching the TS based on random distribution input.

**Figure 2:**
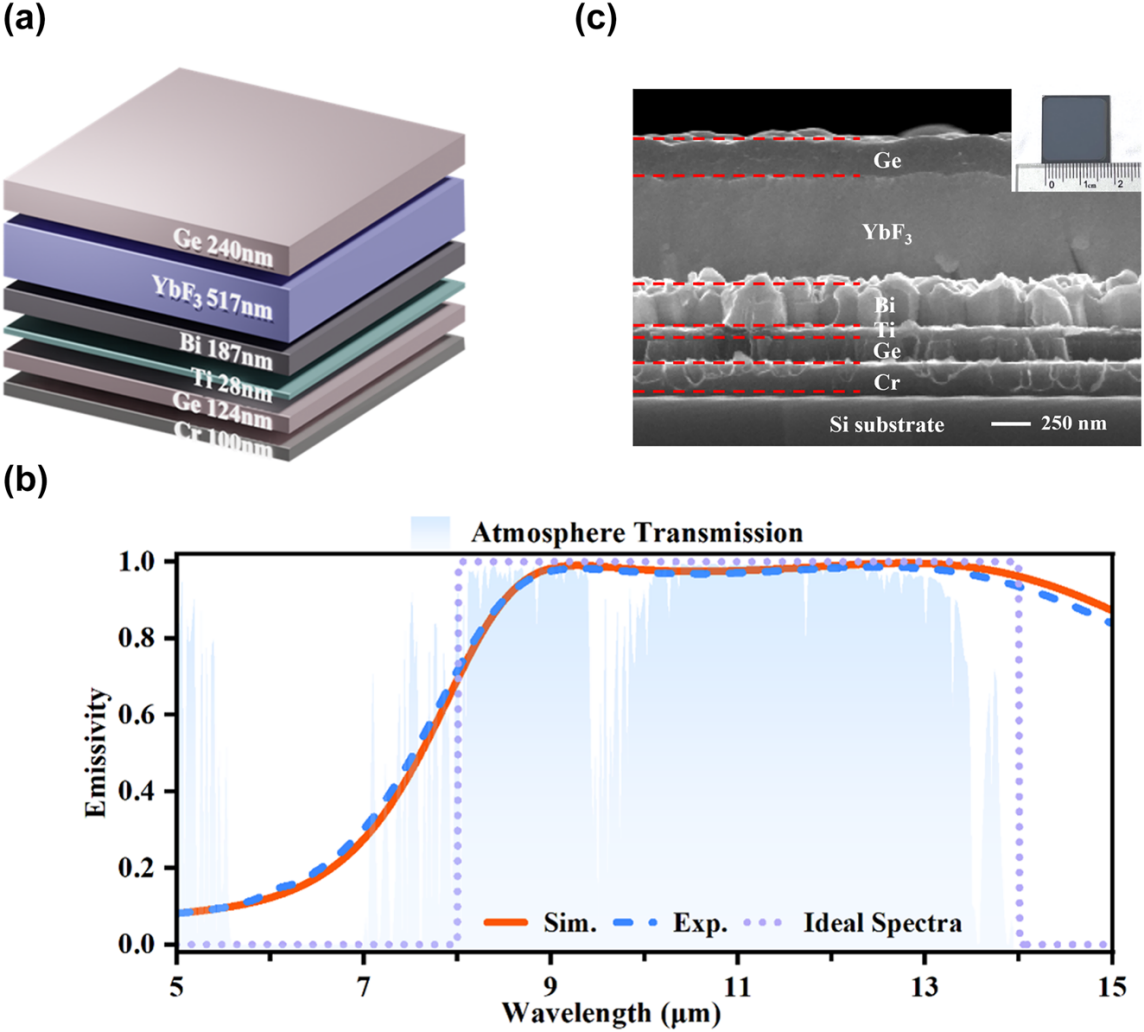
Multilayered metacoating (MMC) for infrared camouflage concept and fabrication. (a) Illustration schematically depicting the proposed MMC. (b) Emissivity spectra for the MMC, where the simulation results (orange line) and experimental measurements (blue dash line) are presented. The comparison includes the ideal high thermal radiation background spectra (purple dot line) and the atmospheric transmission spectra (blue-shaded area). (c) The cross-section SEM and top-view optical images of the experimental sample.

Figure 2(a) illustrates the MMC structure generated by DNN optimizer, comprising six layers with the following composition and thicknesses: Ge (240 nm), YbF_3_ (517 nm), Bi (187 nm), Ti (28 nm), Ge (124 nm), and Cr (100 nm). The theoretical emission spectrum of optimized MMC device depicted by the solid line in Figure 2(b), demonstrates good agreement with the ideal LWIR camouflage spectra, represented by the dotted line. Specifically, the optimized MMC structure exhibits an average emissivity of 0.96 within the LWIR atmospheric transmission window (ATW) and a low average emissivity of 0.25 in other mid-infrared (MIR) bands, thereby achieving the desired spectral characteristics for effective LWIR camouflage.

**Figure 3:**
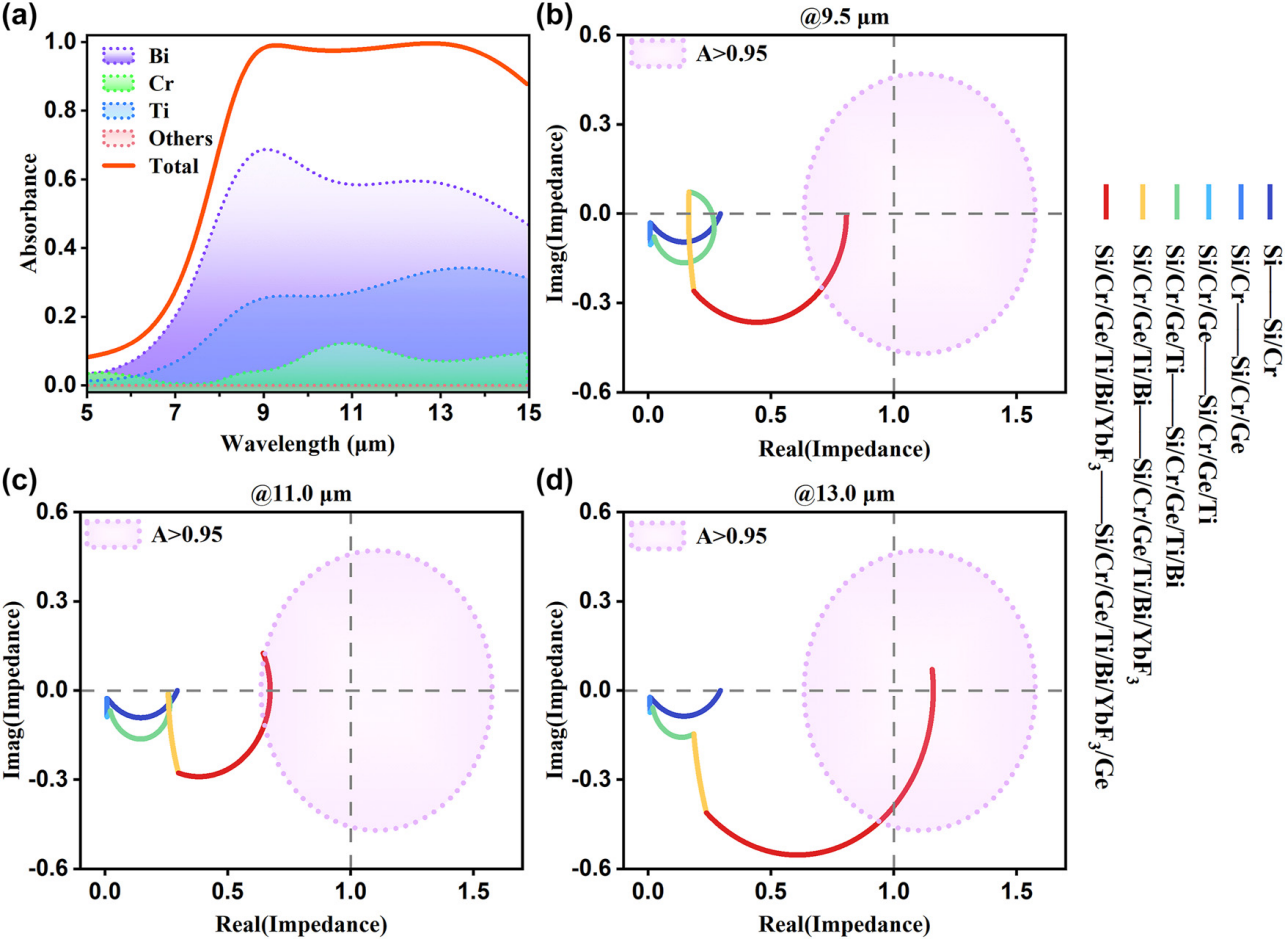
Power dissipation mechanism and optical impedance of the MMC device. (a) The normalized power loss spectra for the total and individual components of the proposed structure. (b–d) The trends of the optical impedance (*Z*) of the MMC layered by layered at the three resonant wavelengths of 9.5, 11.0, and 13.0 μm. The purple short-dotted circles represent the impedances as A > 0.95.

To provide theoretical insights, numerical simulations and calculations were conducted. As shown in Figure 3(a), the normalized power loss spectra for the total and individual components of the MMC device were examined. Most of the power dissipation occurs in the high-loss layers, such as Bi, Ti, and Cr. On average, over 55 % of the incident wave energy is absorbed by the Bi layer in the LWIR band, while the Ti and Cr layers dissipate approximately 30 % and 10 %, respectively. This remarkable energy dissipation is strongly correlated to the unique optical properties of Bi (exhibiting a high refractive index and large absorption coefficient in the MIR band) [46], [47], and Ti (possessing strong optical absorption despite its extremely small thickness). The measured optical properties of these materials are shown in Section S2 of the supplementary Material. We further investigated the mechanism underlying the MMC device’s highly efficient absorption over the LWIR ATW. Since the bottom Cr film is sufficiently thick to prevent light transmission, maximizing absorption involves minimizing reflection, which can be analyzed through optical impedance (Z). The optical impedance Z, used here to analyze the emission properties, is given by the normalized expression *Z* = *Z*_eff_/*Z*_0_, with *Z*_0_ = 377Ω denoting the free-space impedance and *Z*_eff_ representing the absolute impedance of the MMC structure. As illustrated in Figure 3(b–d), the optical impedance locus of the proposed MMC was examined at different resonance wavelengths (see Section S3 of the supplementary material for detailed numerical computations) as the thickness of various layers increased. The point (1,0) on the chart represents perfect absorption, indicating ideal impedance matching to free space. The purple short-dotted circles represent the impedance trajectory where absorption equals 95 %. It was observed that the optical impedance locus of the MMC device, though initially far from the perfect absorption point, consistently falls within the 95 % absorption circle as the structural parameters approach the design configuration at the typical wavelengths (9.5, 11.0, 13.0 μm), indicating that the condition of impedance matching is reasonably well satisfied in the total working band.

**Figure 4:**
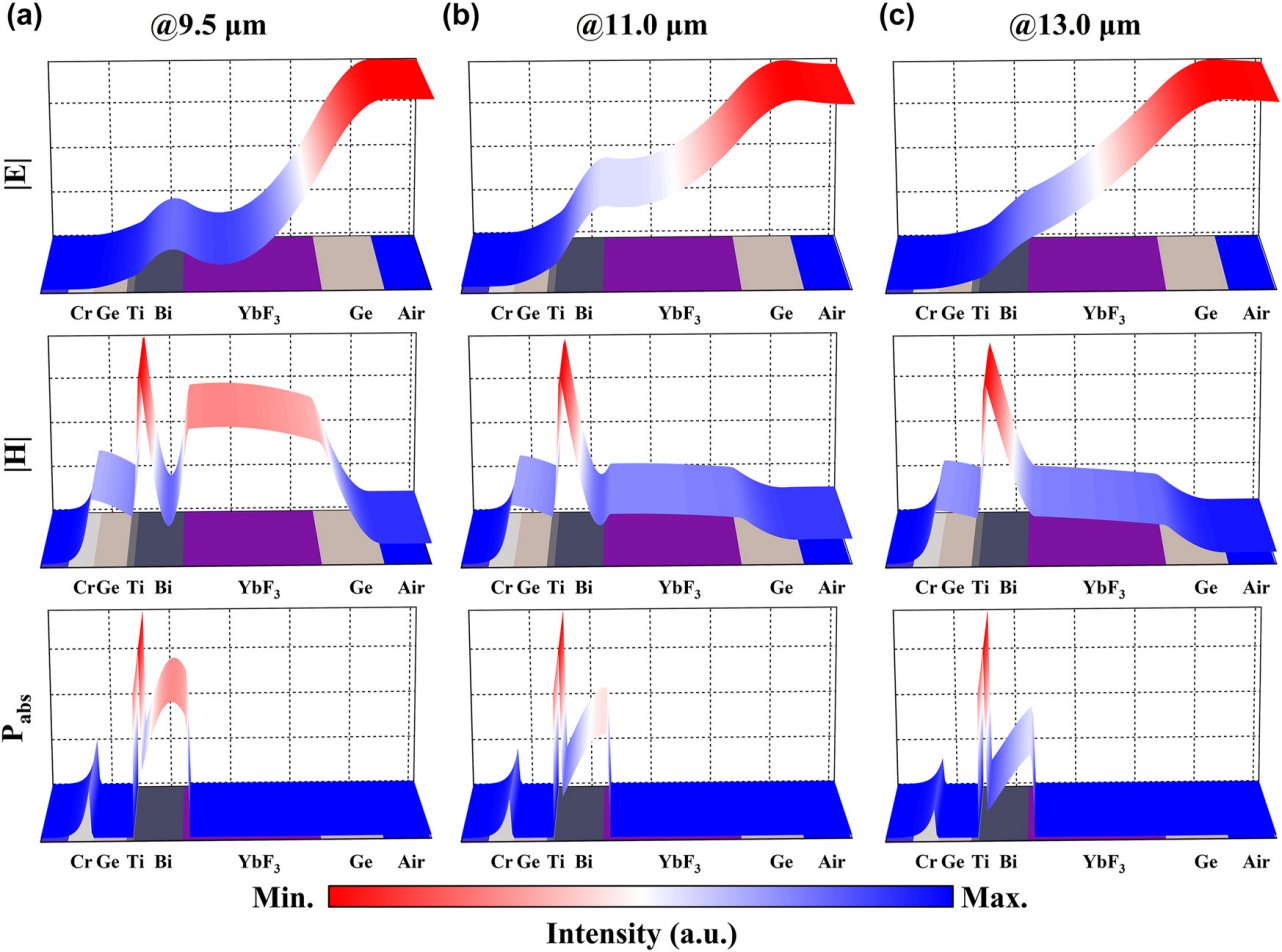
(a–c) Simulated normalized electric field, magnetic field, and time-averaged power dissipation distribution at 9.5 μm, 11.0 μm, and 13.0 μm, respectively.

To gain a more comprehensive understanding of the mechanism behind broadband emission, the finite-difference time-domain (FDTD) method was used to analyze the distributions of electromagnetic fields and energy dissipation within the proposed structure, excited by a plane wave incident perpendicular to the MMC emitter. The simulated results revealed that the magnetic field is concentrated in three distinct regions: the YbF_3_ layer, the interface between the Bi and Ti layers, and the interface of the underlying Ge and Cr layers, while the electric field is predominantly concentrated at the interface between the YbF_3_ and Bi layers. Specifically, Figure 4(a) displays the field distributions at the wavelength of 9.5 μm, where the magnetic field is localized in those aforementioned areas and the electric field is slightly concentrated at the interface between YbF_3_ and Bi. At 11.0 μm, as shown in Figure 4(b), the localization of the magnetic field in the YbF3 layer has diminished, while the other regions maintain a strongly localized magnetic field. Concurrently, the electric field in the interface of YbF_3_ and Bi increases. Moving to the longer wavelength of 13.0 μm, Figure 4(c) indicates that the localization of the magnetic field in the YbF_3_ layer further weakens, and the electric field displays a downward trend inside the structure without obvious localization. These field localizations correspond to the energy dissipation within the high-lossy materials in their vicinity. Figure 4(a–c) reveal that the energy loss within the Bi layer decreases with increasing wavelength, which is attributed to the continuous decrease in the localization of the magnetic field within the nearby YbF_3_ layer. Since the magnetic field maintains a higher level of localization in the other regions, the Ti layer sustains a high absorption, while the absorption of the Cr layer remains relatively stable, consistent with the observations in Figure 3(a). The results indicate that the structure consistently exhibits high energy dissipation characteristics through various forms of electromagnetic field localization across the LWIR spectral region, which is critical for achieving broadband emission. These findings confirm that the broadband emission characteristics depend critically on both the engineered field localization within the precisely optimized MMC’s multilayer structure and the selection of high-loss materials (Bi/Ti). The phenomenon is driven by multiple localized dielectric cavities that confine light, where the adjacent Bi/Ti layers efficiently dissipate the confined energy, resulting in high energy dissipation across a wide LWIR bandwidth. A detailed analysis of the impact of the thickness of each layer of the metacoating on its emission properties is provided in Section S4 of the supplementary material.

To experimentally validate the proposed design, the MMC devices were fabricated using electron beam evaporation, as detailed in the Experimental Section. The measured emissivity spectra of the fabricated devices are presented in Figure 2(b) as short-dashed lines, demonstrating excellent agreement with the theoretical results. This correlation between experimental and theoretical results confirms the guidance of the design optimization process. The morphology of the metacoating was characterized using scanning electron microscopy (SEM), and a wafer-scale sample’s optical image is displayed in Figure 2(c). Notably, the prototype device exhibits remarkable deep-subwavelength characteristics, with a total thickness of only 1,220 nm, which is less than one-tenth of the maximum working wavelength.

**Figure 5:**
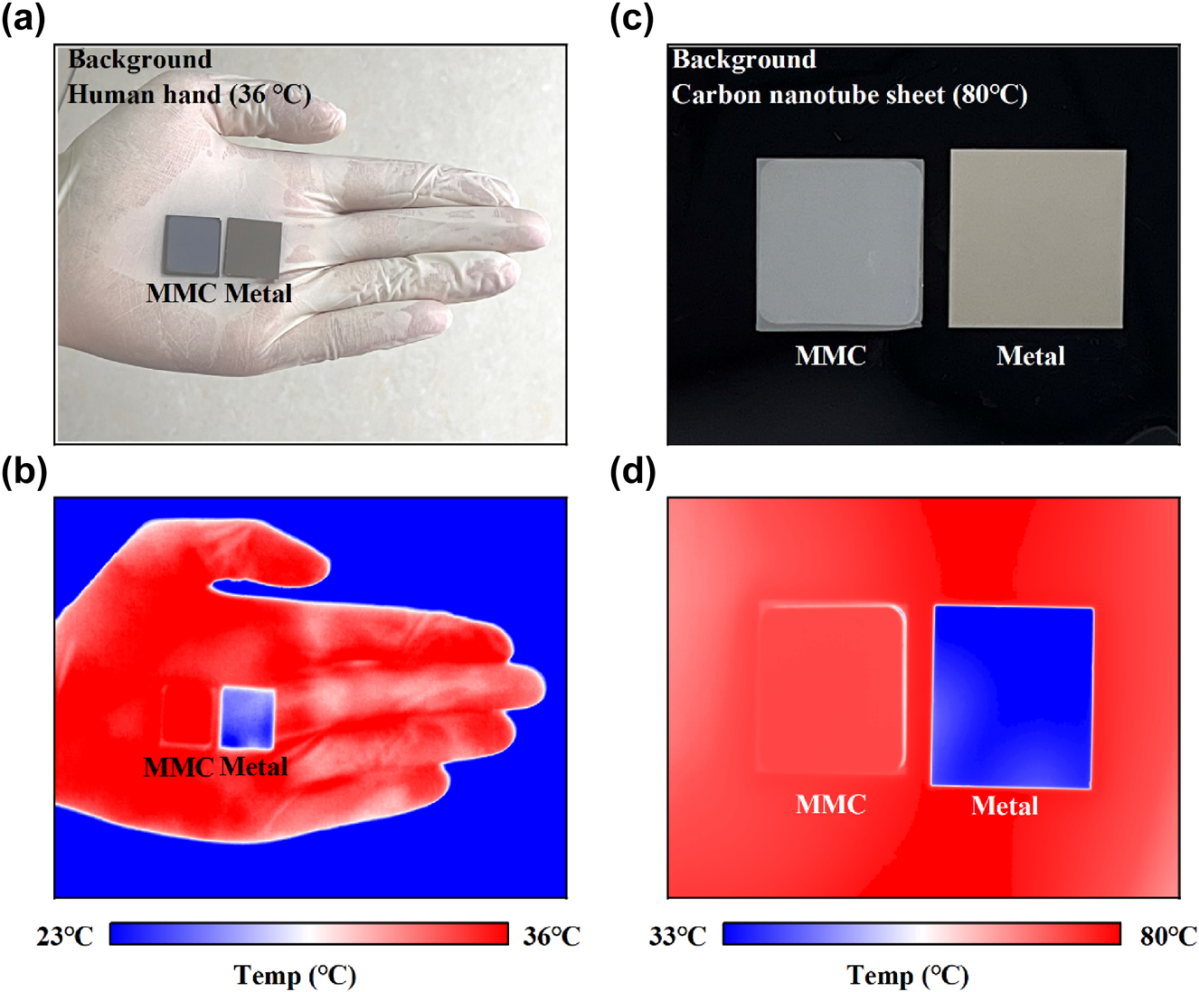
(a) Optical and (b) infrared images of the MC and metal samples placed on the author’s palm. (c) Optical and (d) infrared images of the fabricated samples and reference samples were placed on a carbon nanotube sheet that was mounted on a heating stage and the temperature was set as 80 °C.

To evaluate the thermal infrared camouflage performance of the proposed MMC emitter, two distinct experimental setups were employed. In the first experiment, the MMC emitter and a metallic reference sample (a 200 nm Cr film) that has low emissivity across LWIR spectral region were positioned on the author’s palm side by side, as confirmed by the optical image in Figure 5(a). The thermal emission profile was recorded with an LWIR camera (8–14 μm) under ambient temperature conditions, with results depicted in Figure 5(b). The metallic reference sample displayed strong infrared contrast against the palm background due to its distinct thermal radiation properties. In striking contrast, the MMC emitter achieved exceptional infrared camouflage, becoming virtually indistinguishable from the palm background. This remarkable performance stems from the precisely engineered thermal emission characteristics of the MMC emitter, which closely mimics the high emissivity of the human palm.

The second experiment involved placing the samples on a carbon nanotube sheet mounted on a heated stage maintained at 80 °C. The carbon nanotube substrate is distinguished by its broadband super-black behavior, with detailed emissivity and radiance properties provided in Section S5 of the supplementary material. Figure 5(c) and (d) present the optical and LWIR images of this case, respectively. While the metallic reference sample remained discernible in the infrared image, the MMC emitter demonstrated superior camouflage performance against the high-emissivity carbon nanotube background. The experimental validation involved two distinct high-emission backgrounds to rigorously assess the MMC emitter’s camouflage capabilities. In the first scenario, the MMC sample became virtually indistinguishable from a human palm (*ε* < 1), a common real-world background with moderate emissivity. This exceptional performance confirms that the MMC’s precisely engineered thermal emission successfully mimics the spectral characteristics of natural skin. In the second scenario, the sample was evaluated against a carbon nanotube (CNT) sheet – a near-blackbody surface with broadband, high emissivity. The MMC emitter again demonstrated superior camouflage, seamlessly blending into the high-emissivity background, whereas the metallic reference remained clearly visible. Both these experiments conclusively validate the MMC emitter’s practical applicability for infrared camouflage.

**Figure 6:**
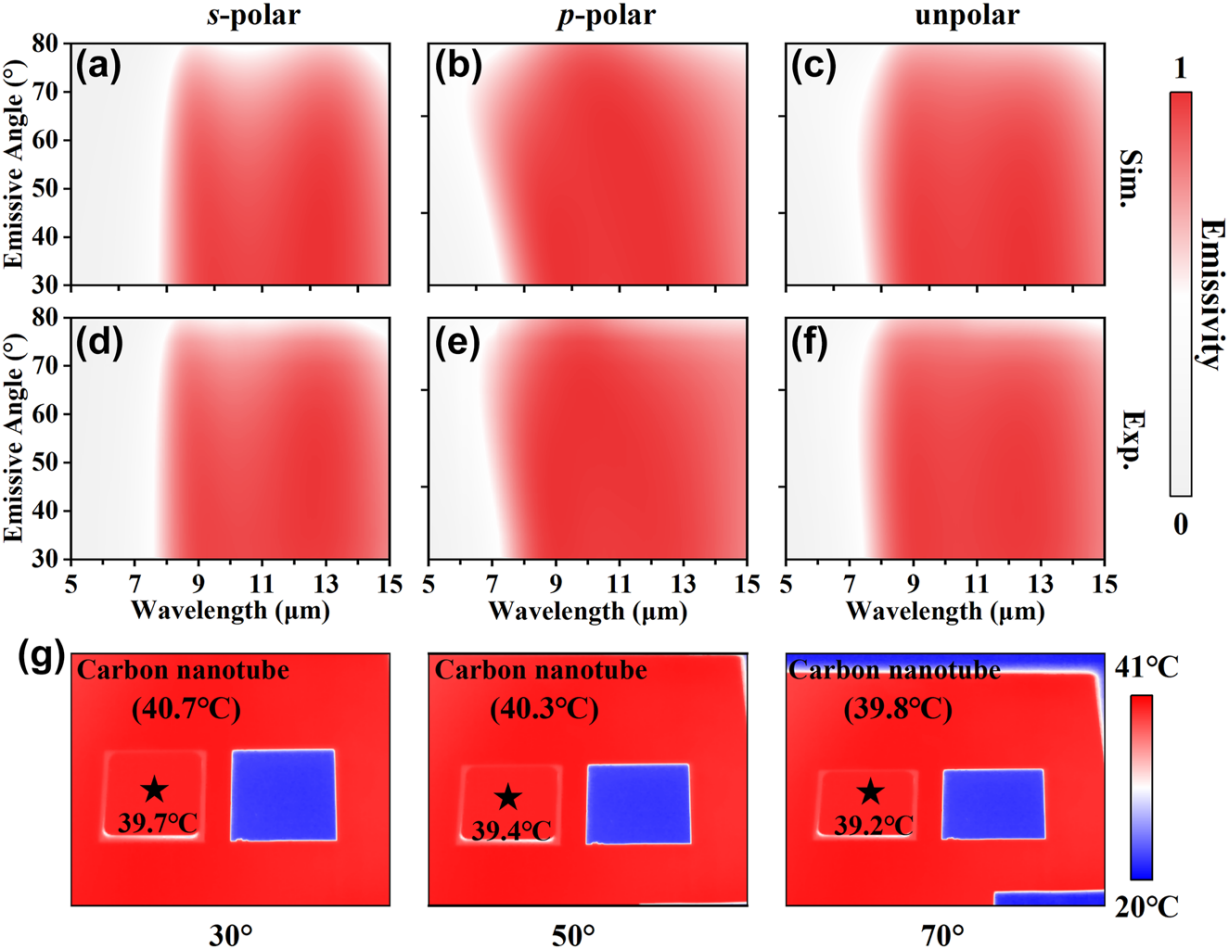
(a–c) Simulated and (d–f) experimental measured spectra as a function of wavelengths and incident angles for *s-*, *p-*, and unpolar light, respectively. (g) Infrared images of the MMC and metal samples placed on the carbon nanotube captured at different observation angles (30°, 50°, 70°).

The angular performance of the proposed MMC emitter was comprehensively characterized through measurements spanning an angular range of 30–80°. Figure 6(a–c) illustrate the experimental emissivity as functions of wavelength and incident angle for *s*-polarized, *p*-polarized, and unpolarized light. The results reveal the emitter’s robust angular performance, sustaining an average LWIR emissivity exceeding 0.9 up to a 70° incidence angle across all polarizations. The theoretical spectra, calculated via the transfer matrix method (TMM), are displayed in Figure 6(d–f), exhibiting excellent consistency with the experimental findings. The MMC emitter’s angle-independent emission behavior is attributed to its deep-subwavelength thickness of merely 1.2 μm, a dimension substantially smaller than the wavelengths in the LWIR region. Additionally, its impedance matching property, detailed in Section S6 of the supplementary material, further contributes to this phenomenon. To underscore the practical significance of this angular insensitivity, Figure 6(g) presents infrared images captured in the second experimental scenario (heated carbon nanotube sheet background) at various observation angles. Notably, even at a large oblique angle of 70°, the MMC emitter retains its effective camouflage capability, seamlessly blending into the high-radiation background. This further demonstrates the emitter’s potential for real-world thermal infrared camouflage applications.

## 3 Conclusions

In conclusion, this work has proposed and experimentally demonstrated a broadband, lithography-free, ultra-thin, high-performance metacoating emitter for LWIR camouflage applications in high-emission environments. The optimized MMC emitter was developed through an automated framework empowered by a DNN optimizer. The resulting emitter exhibits an exceptional average emissivity exceeding 0.96 across the LWIR ATW, while maintaining a low emissivity below 0.25 in the other MIR spectral region, as validated by both experimental and theoretical analyses. The outstanding thermal emission characteristics of the MMC emitter can be primarily attributed to its broadband impedance matching design, which facilitates efficient energy dissipation through localized EM field confinement within the multi-layer structure, particularly in the proximity of high-lossy Bi and Ti components. Furthermore, IR imaging experiments were conducted to directly showcase the superior camouflage performance of the proposed emitter against highly radiative backgrounds. Importantly, the deep-subwavelength thickness (1.22 μm) of the MMC structure confers angle-independent emission properties, with the favorable emissive behavior (∼0.9 emissivity) being maintained even at large incidence angles up to 70°. This angular robustness would be an asset in real-world thermal IR camouflage applications, as it enables effective concealment against modern detection methods employing wide-angle and polarization-sensitive measurements. The findings of this work indicate the significant potential of the developed MMC emitter for advanced IR camouflage applications.

## 4 Materials and methods

### 4.1 Optical constants measurement

The optical constants of the deposited layers Cr, Ge, Ti, Bi, and YbF_3_ were measured and fitted in the infrared range using infrared spectroscopic ellipsometry (Sendira Sentech, 1,500–25,000 nm) and the optical constants of Au, Ag, Al, Ti, MgF_2_, Si, ZnS, SiO_2_, HfO_2_ were obtained from literature [50].

### 4.2 Numerical computations and simulations

The numerical computations were carried out through transfer matrix methods (TMM). Electromagnetic wave numerical simulations were conducted based on finite-difference-time-domain (FDTD) method. In the simulations, a plane source was launched into a two-dimensional FDTD simulation zone. Periodic boundary conditions were imposed on the *x* axes, and perfect matched layer (PML) were imposed on the *y* axes.

### 4.3 Sample fabrication and characterization

The samples and reference sample were fabricated by electron-beam evaporation (Syskey Technology UHEB-LC6-03) based on layer-by-layer deposition process. The Ge, YbF_3_, Bi, Ti and Cr layers were deposited at room temperature at rates of 0.3 nm/s, 0.6 nm/s, 0.2 nm/s, 0.2 nm/s and 0.1 nm/s, respectively. The deposition rates were monitored by gold coated crystals (Inficon) and the chamber pressure were maintained at ∼10-6 Torr (Pfeiffer) during the deposition process. The cross-section of the sample was characterized by scanning electron microscopy (FEI Sirion 200).

The angle-resolved reflectance spectra over a range of 30–80° were obtained using a Fourier transform infrared spectrometer (Thermo Scientific Nicolet iS50), both covering a spectral range of 8,000–400 cm^−1^. Additionally, the morphologies of the proposed MMC were characterized using a scanning electron microscope (FEI Sirion 200) with an accelerating voltage of 10 kV. Thermal measurements were conducted using an LWIR camera (Fotric 220) with a default emissivity of 0.95, operating in the 8–14 μm wavelength range.

## Supplementary Material

Supplementary Material Details
