# Supplementary material for: Lithography-free subwavelength metacoatings for high thermal radiation background camouflage empowered by deep neural network
Source: Nanophotonics. 2025 Dec 8;14(27):5291–300. doi: 10.1515/nanoph-2025-0409 (PMC12717922; doi:10.1515/nanoph-2025-0409)
Supplement: Supplementary file 1 — Supplementary Material Details [file j_nanoph-2025-0409_suppl_001.docx]

Supplementary material

Lithography-free subwavelength metacoatings for high thermal radiation background camouflage empowered by deep neural network

Qianli Qiu^1, 4^, Kang Li^2, 3^, Dongjie Zhou^1, 4^, Yuyang Zhang^2^, Jinguo Zhang^2^, Zongkun Zhang^1, 4^, Yan Sun^1, 4, *^, Lei Zhou^3^, Ning Dai^1, 5^, Junhao Chu^1, 2^, and Jiaming Hao^2, *^

^1^ State Key Laboratory of Infrared Physics, Shanghai Institute of Technical Physics, Chinese Academy of Science, Shanghai 200083, China

^2^ Institute of Optoelectronics & College of Future Information Technology, Shanghai Frontiers Science Research Base of Intelligent Optoelectronics and Perception, and State Key Laboratory of Photovoltaic Science and Technology, Fudan University, Shanghai 200433, China

^3^ Shanghai Key Laboratory of Metasurfaces for Light Manipulation, State Key Laboratory of Surface Physics, Key Laboratory of Micro and Nano Photonic Structures (Ministry of Education) and Department of Physics, Fudan University, Shanghai 200433, China

^4^ University of Chinese Academy of Sciences, No. 19A Yu Quan Road, Beijing 100049, China

^5^ Hangzhou Institute for Advanced Study, University of Chinese Academy of Sciences, Hangzhou 310024, China

*Corresponding author: [sunny@mail.sitp.ac.cn](mailto:sunny@mail.sitp.ac.cn); [jmhao@fudan.edu.cn](mailto:jmhao@fudan.edu.cn)

**Contents**

[**Section S1 The Optimized process of MLP framework 3**](#_Toc196854974)

[**Section S2 Experimental Optical Properties of Each Layers in MMC 5**](#_Toc196854975)

[**Section S3 Transfer matrix methods for multilayered structure calculation and impedance analysis 6**](#_Toc196854976)

[**Section S4 Impact of the thickness of metacoating on its emission property 9**](#_Toc196854977)

[**Section S5 Emissivity calculation for the MMC, Carbon Nanotube, and Metal 11**](#_Toc196854978)

[**Section S6 The analysis of angular and polarization dependence by impedance matching condition 13**](#_Toc196854979)

Section S1 The Optimized process of MLP framework


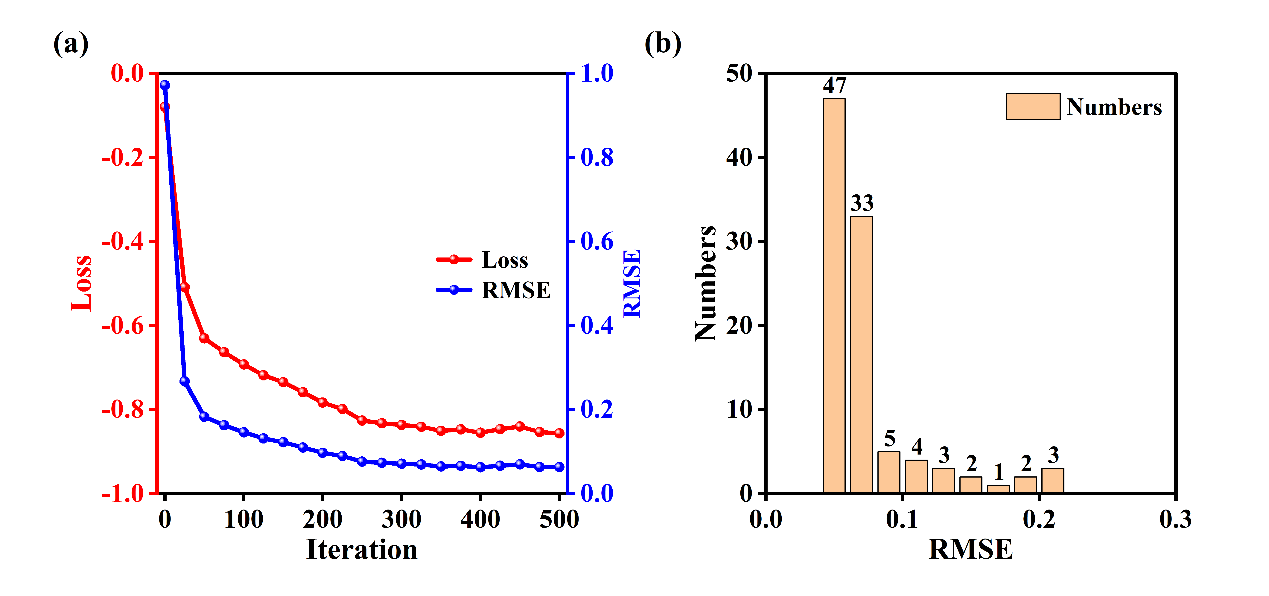


**Figure S1.** (a) Training convergence profile showing progressive loss and root mean square errors (RMSE) minimization over 500 iterations. (b) Histogram distribution of RMSE between MLP-generated metacoating designs and the target ideal spectrum across 100 test samples.

The proposed methodology integrates a Multilayer Perceptron (MLP) with the Transfer Matrix Method (TMM) to establish an optimization framework that eliminates conventional training dataset requirements. As illustrated in Figure 1, this synergistic approach enables direct learning of optimal parameter distributions from TMM-generated electromagnetic simulation data through training process.

The MLP architecture employs a 260-512-1024-512-260 configuration with three hidden layers, accepting random noise matrices x ∈ ℝ^(N×M) as inputs. Here, N=2048 denotes the batch size for ensemble metacoating generation, while M=260 represents the total optimization parameters derived from 20 structural layers (Ls=20) with 13 material options per layer (Ms=13). Through a softmax-activated output layer, the network transforms input noise into normalized design parameters y ∈ [0,1]^(N×M). These parameters are subsequently reshaped into Ls×Ms matrices, where each row specifies layer thickness and material composition via one-hot encoding.

The physical realization module converts these numerical outputs into metacoating configurations for spectral evaluation using TMM simulations across 3-14 μm wavelengths. A custom loss function quantifies spectral deviations from the target 8-13 μm ideal response, driving Adam optimization (β₁=0.9, β₂=0.99, weight decay=0.001) through backpropagation. This iterative process progressively aligns MLP-generated designs with desired spectral characteristics.

Following 500 training iterations, the converged network demonstrates robust design capabilities, as shown in Figure. S1(a). Post-training evaluation with a test batch size of 100 reveals that >85% of generated metacoating structures achieve spectral root mean square errors (RMSE) <0.1 relative to the ideal profile in Figure S1(b). This performance confirms the network's ability to automatically synthesize metacoating designs with optimized thickness distributions and material selections through inverse design principles.

Section S2 Experimental Optical Properties of Each Layers in MMC


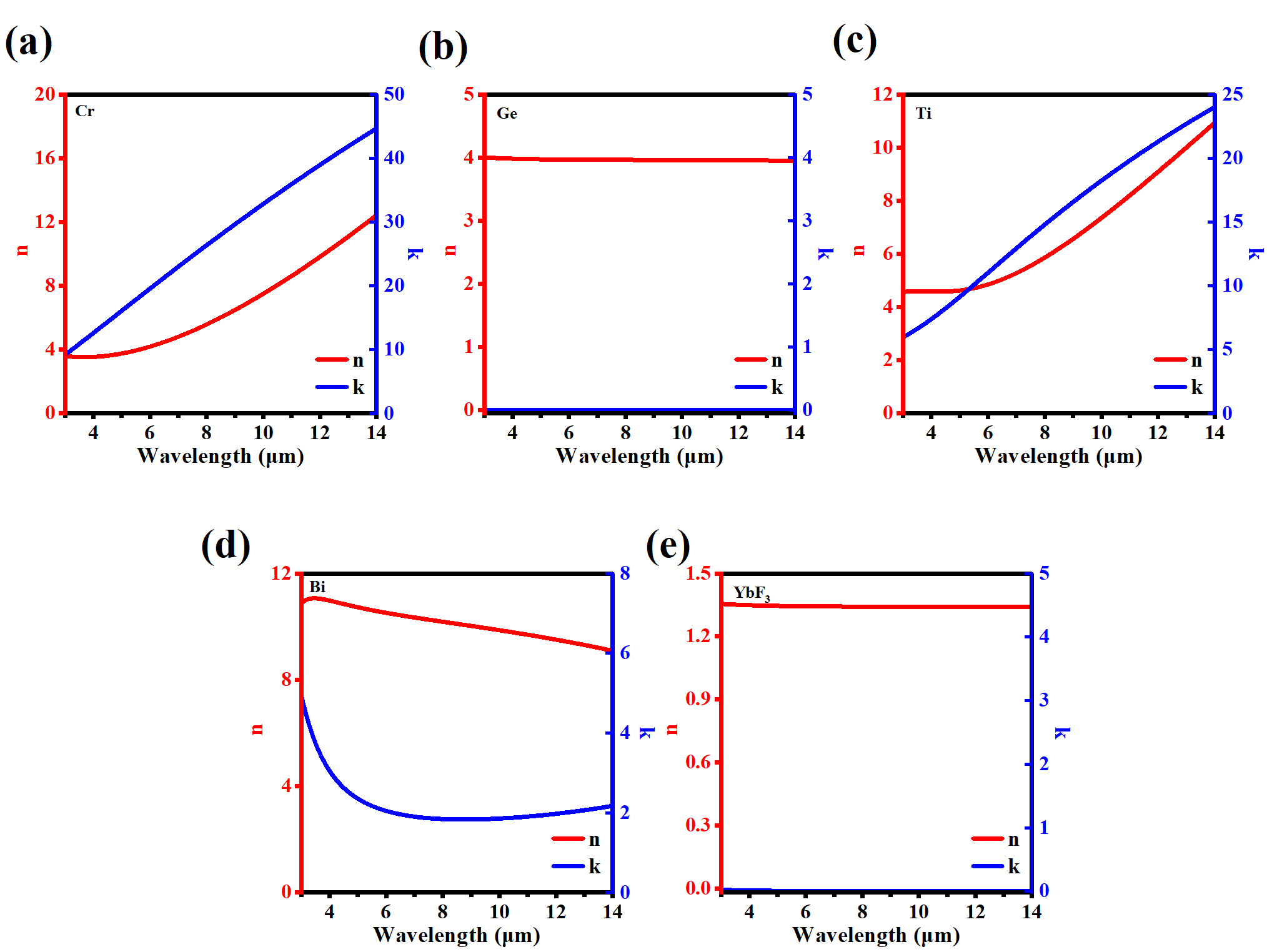


**Figure S2.** (a-e) Experimental optical properties measured by ellipsometry of each layers in MMC (Cr, Ge, Ti, Bi, YbF₃) contain real and imaginary parts of refractive index in 3-14 μm.

To achieve a more accurate alignment between experimental and simulation results, we undertook the growth of monolayer films for each material (Cr, Ge, Ti, Bi, YbF₃) constituting the MMC structure. Subsequently, we employed ellipsometry to characterize their optical properties within the infrared spectrum spanning 3-14 μm. The optical parameters of these materials, which served as the foundation for the subsequent simulation calculations, are presented in Figure S2.

Section S3 Transfer matrix methods for multilayered structure calculation and impedance analysis

The spectral response of the multilayered metacoating was rigorously analyzed through numerical implementation of the Transfer Matrix Method (TMM). This computational approach models electromagnetic wave propagation through stratified media by constructing characteristic matrices that relate input and output field components. For an m-layer system, the transfer matrix formalism is mathematically expressed as:

$$\binom{E_{in}/E_{out}}{H_{in}/E_{out}}=\binom{M_{1}}{M_{2}}=\{\prod_{i=1}^{m} [\begin{matrix} cos\delta_{i} & \frac{isin\delta_{i}}{\eta_{i}} \\ i\eta_{i}sin\delta_{i} & cos\delta_{i} \end{matrix}]\}\binom{1}{\eta_{sub}}$$

$\delta_{i}=\frac{2\pi n_{i}d_{i}cos\theta_{i}}{\lambda}$, $\eta_{i}=n_{i}cos\theta_{i}$ (TE-wave), $\eta_{i}=\frac{n_{i}}{cos\theta}$ (TM-wave)

where $n_{i}, \theta_{i}, d_{i}$ represent the complex refractive index, incident angle, and layer thickness in the *i*-th layer, respectively. The reflectance and transmission of this system:

$$R=\left( \frac{\eta_{0}M_{1}-M_{2}}{\eta_{0}M_{1}+M_{2}} \right)\left( \frac{\eta_{0}M_{1}-M_{2}}{\eta_{0}M_{1}+M_{2}} \right)^{*},T=\frac{4\eta_{0}Re(\eta_{sub})}{\left( \eta_{0}M_{1}+M_{2} \right)\left( \eta_{0}M_{1}+M_{2} \right)^{*}}$$

The emissivity/absorption spectrum is then obtained by:

$E\left( \lambda\right)=A\left( \lambda\right)=1-R(\lambda)-T(\lambda)$.

We implemented the transmission line model (TL model) to quantify the impedance matching conditions for near-perfect absorption in our metacoating system. The reflection coefficient was analytically derived as:

$$r=\frac{Z_{out}-Z_{in}}{Z_{in}+Z_{out}}$$

$Z_{out}$*,* $Z_{in}$ represent the output impedance and input impedance, respectively, to analyze this condition. Incident light propagates from free space to our absorber, and the characteristic impedance of free space, represented by $Z_{0}$, is a constant value of 377 Ω. Therefore, if the equivalent impedance of our structure is closer to the characteristic impedance of free space, it is more likely to achieve near-perfect absorption at the corresponding wavelength. The characteristic matrix of TMM calculation as complied, which provides us with the equivalent impedance of our structure, corresponding to

$$Z_{eff}= \frac{E_{out}}{H_{out}}=\frac{M_{2}}{M_{1}}$$

The impedance rings (E=A=0.95), are represented by their trajectory equation about *R* and *L*, where *R* and *L* represent the real and imaginary parts of the impedance, expressed as the following equation.

$\left( 1-A \right)=\left| \frac{Z_{0}-\left( R+jL \right)}{Z_{0}+\left( R+jL \right)} \right|^{2}$

Section S4 Impact of the thickness of metacoating on its emission property


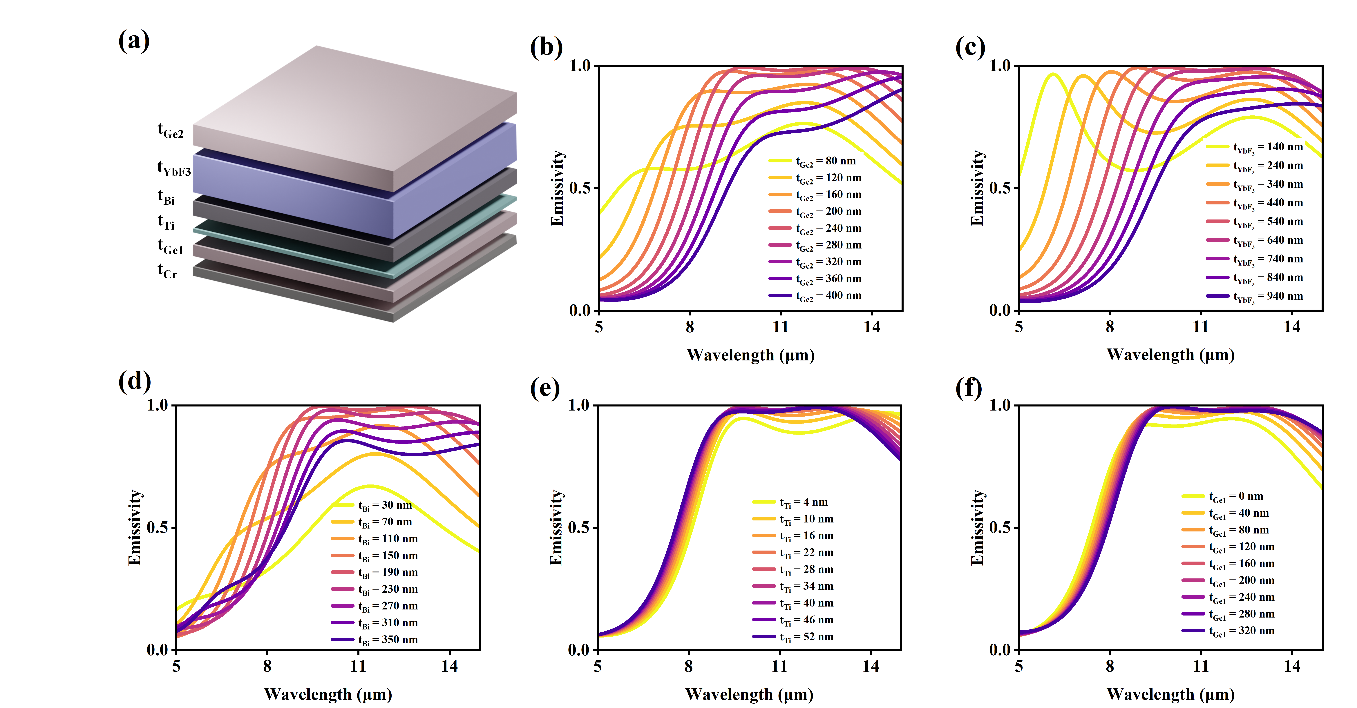


**Figure S3.** (a) Illustration schematically depicting the proposed metacoating. (b) Simulated emission spectra as a function of wavelengths and Ge thickness (t_Ge2_), (c) YbF_3_ thickness (t_YbF3_), (d) Bi thickness (t_Bi_), (e) Ti thickness (t_Bi_), (f) Ge thickness (t_Ge1_).

We investigated the influence of individual layer thickness variations on the spectral characteristics of the metacoating. Specifically, as the Ge film thickness (t_Ge2_) exceeds its optimized value, the metacoating exhibits a progressive redshift in its primary emission peak accompanied by degradation of its broadband emission characteristics. This thickness deviation induces a transition from broadband emission to a dominant single resonance peak beyond 14 μm (Figure S3(b)).

Similar thickness sensitivity was observed for the YbF_3_ layer (t_YbF3_). Figure S3(c) demonstrates that significant reduction of YbF_3_ film thickness relative to its optimal value disrupts the multi-resonance coupling mechanism, resulting in pronounced resonance peaks near 5 μm rather than maintaining the characteristic broadband emission profile.Thickness modulation of the Bi layer (t_Bi_) showed distinct behavior. As shown in Figure S3(d), substantial reduction below the optimal thickness degrades the emission spectrum to a narrow resonance peak centered at 11 μm. In contrast, simultaneous thickness variations of the Ti (t_Ti_) and Ge_1_ (t_Ge1_) layers (Figure S3(e-f)) exhibited minimal impact on both the structural emission spectra and the metacoating's emissivity across the 8-13 μm spectral range. This insensitivity suggests that these layers primarily function as structural components rather than active resonators.

The comparative analysis confirms that the metacoating's exceptional broadband selective emission originates from constructive interference between independently coupled resonance modes within the hierarchical thin-film system. Such decoupled resonance dynamics enable simultaneous preservation of high emissivity and broadband spectral selectivity across the LWIR region.

Section S5 Emissivity calculation for the MMC, Carbon Nanotube, and Metal


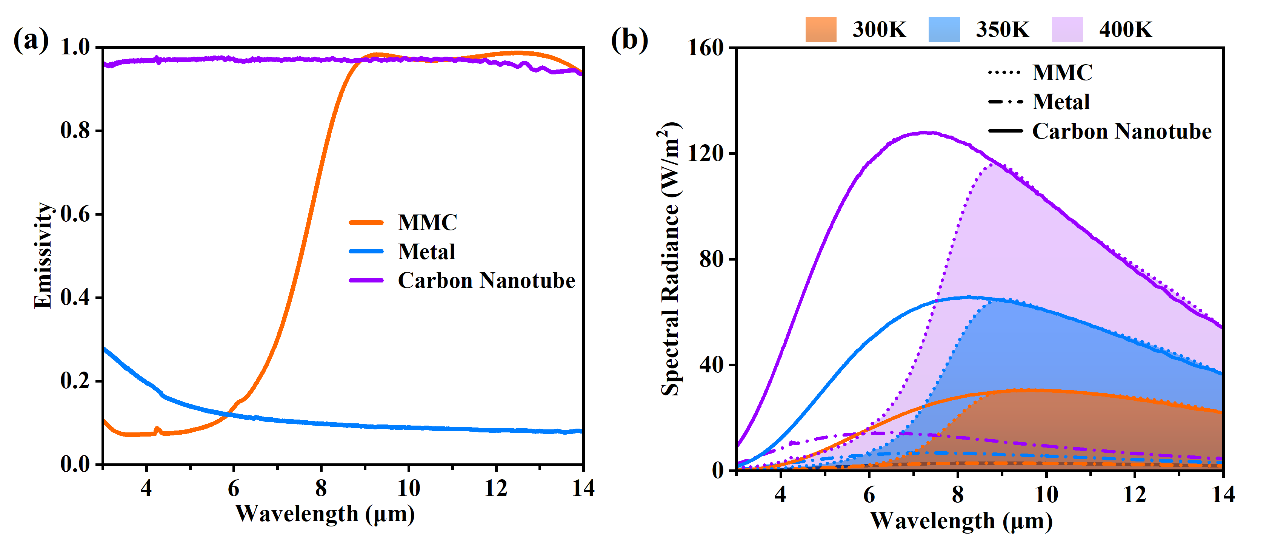


**Figure S4**. (a) Measured emissivity spectra of our MMC sample, carbon nanotube, and metal. (b) Spectral radiation of MMC sample, carbon tube, and metal at 300K, 350K, and 400K.

The formula that we use to calculate the spectral radiation intensity of our MMC carbon tube, and metal as below:

$$P_{rad}\left( \lambda,T \right)=\varepsilon_{s}\left( \lambda,T \right)\cdot M_{bb}(\lambda,T)$$

The average emissivity of an object from $\lambda_{1}$ to $\lambda_{2}$ can be calculated using the following formula:

$$\bar{\varepsilon}_{\lambda_{1}-\lambda_{2}}=\frac{\int_{\lambda_{1}}^{\lambda_{2}} \varepsilon\left( \lambda\right)\cdot M_{bb}\left( \lambda, T \right) d\lambda}{\int_{\lambda_{1}}^{\lambda_{2}} M_{bb}\left( \lambda, T \right) d\lambda}$$

Here, *ε*(𝜆) represents the emissivity of the sample, which can be obtained from the measured absorption spectrum, and 𝑀(𝜆,𝑇) denotes the blackbody radiation as described by Planck’s Law. Consequently, the average emissivity is dependent on temperature. At room temperature, the LWIR average emissivity of the MMC sample, carbon nanotube, and metal calculated from the measured spectrum, as shown in Figure S4(a), are 0.96, 0.96, and 0.09.

Furthermore, using these equations and the emissivity data measured by FTIR, we calculated the spectral radiation intensity of the samples, presented in Figure S4(b). Table S1 displays their emissions across different bands at various temperatures.

| **LWIR Emission(W/m^2^)**  **8-13 μm** | **300 K** | **350 K** | **400 K** |
| --- | --- | --- | --- |
| **MMC** | 165.7 | 320.2 | 531.0 |
| **Carbon Nanotube** | 166.5 | 332.94 | 537.1 |
| **Metal** | 15.0 | 29.1 | 48.6 |
| **Ideal Blackbody** | 172.8 | 335.0 | 556.8 |

**Table S1.** The radiance power of MMC sample, carbon nanotube, and metal at various temperatures across different waveband.

Section S6 The analysis of angular and polarization dependence by impedance matching condition


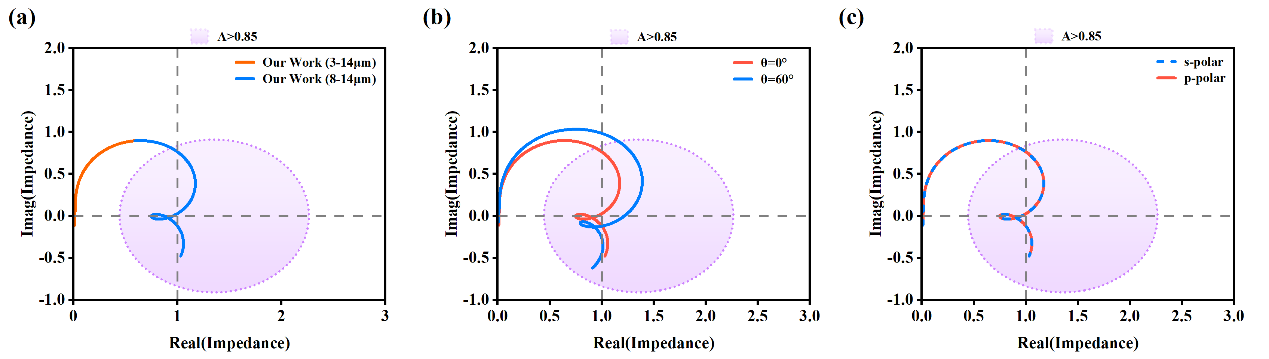


**Figure S5** (a) The optical impedance (Z) of the MMC across the wavelength range of 3-8 μm (orange line) and 8-14 μm (blue line) (b) The MMC’s impedance with different incident angles 0°(orange line) and 60° (blue line), (c) with different polarized incident light p-polar (orange line) and s-polar (blue dash line). The short-dotted circles range indicate the impedance values at A = 0.85.

To further investigate the impedance characteristics of the MMC structure within the mid - infrared band of 3-8 μm, we computed the wavelength - impedance variation, with the results depicted in Figure S5(a). It is evident that across the 8-14 μm broadband range, the structure's impedance predominantly resides within the A > 0.85 impedance ring, highlighting its excellent broadband impedance - matching capability.

The theoretical basis for the angle and polarization insensitivity of our metacoating lies in the impedance - matching conditions. As shown in Figure S5(b), our calculations of the effective impedance at incident angles of 0° and 60° reveal that the MMC structure retains robust impedance - matching performance even at large angles. This characteristic explains its broad absorption features that remain relatively stable under varying angles of incidence. Additionally, Figure S5(c) illustrates the equivalent impedance of the MMC under different polarized light conditions. The data demonstrate that the equivalent impedance exhibits minimal fluctuation across diverse polarization states, theoretically confirming the structure's strong polarization insensitivity.
